# Symptoms of anxiety/depression is associated with more aggressive inflammatory bowel disease

**DOI:** 10.1038/s41598-021-81213-8

**Published:** 2021-01-14

**Authors:** Xin Gao, Yu Tang, Na Lei, Ying Luo, Pingrun Chen, Chang Liang, Shihao Duan, Yan Zhang

**Affiliations:** Department of Gastroenterology, West China Hospital of Sichuan University, Chengdu, People’s Republic of China

**Keywords:** Psychology, Health care

## Abstract

Studies have demonstrated that inflammatory bowel disease (IBD) patients are at an increased risk of developing anxiety and/or depression. IBD patients with depression/anxiety have higher rates of hospitalization and increased disease severity than those without. So far, there is a paucity of data concerning the impact of anxiety/depression on Chinese IBD patients. The aim of this study was to find out the prevalence of symptoms of anxiety/depression in Chinese IBD population and its impact on IBD-related features. This is a cross-sectional study from the southwest China IBD referral center. Eligible participants were divided into those with symptoms of anxiety/depression and those without based on the Hospital Anxiety and Depression Scale (HADS). Demographic data and disease duration, IBD-related surgery, tobacco use, extra-intestinal manifestations, disease activity scores, endoscopic evaluation, laboratory data and current medication use were compared between two groups. A total of 341 IBD patients [221 Crohn’s disease (CD) and 120 ulcerative colitis (UC)] were included. The prevalence of symptoms of anxiety/depression in IBD was 33.1%. CD patients with symptoms of anxiety/depression tended to have higher scores of simple endoscopic scores for Crohn’s disease (SES-CD) (p = 0.0005). UC patients with symptoms of anxiety/depression had a significantly higher Mayo score (p = 0.0017) and ulcerative colitis endoscopic index of severity (UCEIS) (p < 0.0001) than their non-anxiety/depression counterparts. CD-related surgery (p = 0.012) and Crohn's disease activity index (CDAI) (p < 0.0001) were identified as independent risk factors for symptoms of anxiety/depression in CD, while corticosteroid use (p = 0.036) as an independent risk factor for symptoms of anxiety/depression in UC. This study helps our understanding of the prevalence of symptoms of anxiety/depression in IBD patients and its impact on IBD course and reminds us to pay more attention on IBD management with anxiety/depression.

## Introduction

In 1970s, CD patients were first found to be significantly more anxious, neurotic and introverted than both the test norms and the non-psychosomatic medical out-patients and did not differ appreciably from the psychosomatic patients in these respects^1^. Since then, several studies have demonstrated that IBD was associated with psychiatric disorders. The prevalence of anxiety and depression in IBD was at least twice that of the general population^2,3^. Up to 34.7% of IBD patients in active stage were found to have comorbid depression compared with 19.9% of inactive IBD patients^2^. IBD patients with anxiety and depression are therapeutically more demanding and consuming more healthcare resources^4^. Patients with history of depression were more likely to develop IBD, which could be selectively protected by certain antidepressant treatments^5^.

However, the above observations and findings were mainly based on IBD populations from western countries. With the urbanization and westernization, the incidence and prevalence of IBD are increasing in China^6–8^. A systematic review and meta-analysis showed that the summary incidence rate of IBD in China was 1.74 per 100,000 person years, and the corresponding incidence rates of CD and UC were 0.4 and 1.18 per 100,000 person years, respectively^8^. Meanwhile, a recent national survey revealed that the prevalence of psychiatric diseases in the Chinese population is on the rise, with the life time prevalence rates of anxiety and depressive disorders up to 7.6% and 6.8% respectively^9^. IBD and mental health concerns impose huge burden on their sufferers physically, mentally, and financially^9,10^. To our knowledge, few studies have focused on the comorbidity of anxiety/depression in Chinese IBD patients. Thus, the present study aimed to determine the prevalence of symptoms of anxiety/depression in Chinese IBD population and comprehensively analyze the impact of symptoms of anxiety/depression on IBD-related features.

## Methods

### Study population

This is a cross-sectional study. Patients with established diagnosis of CD or UC between September 2017 and December 2019 in West China Hospital of Sichuan University, the largest IBD center in the southwest China were included. The inclusion criteria included: (1) meet the standard criteria for CD or UC, which is based on a combination of clinical, biochemical, stool, endoscopic, cross-sectional imaging, and histological investigations; (2) hospitalized IBD patients, who were willing to participate in our study and could complete the questionnaires. The indication for hospitalization included surgery, disease flare or treatment adjustment. Patients met any one of the following criteria were excluded: (1) concomitant with other psychiatric diseases (other than depression or anxiety disorders) or dementia; (2) some severe chronic diseases, like chronic heart failure and chronic obstructive pulmonary disease, other immune disorders and cancer, which may also cause depression; (3) pregnancy;(4) individuals without full capacity of civil conduct. If a patient may require hospitalization more than once, this patient was only included in the study once.

The diagnosis of CD and UC was based on the third European Evidence-based Consensus on Diagnosis and Management of Crohn’s disease and Ulcerative Colitis^11,12^. Anxiety and depression symptoms were determined using the Hospital Anxiety and Depression Scale (HADS) questionnaire. Patients completed the questionnaire at the time of the first clinical encounter recorded within this study period with the help of doctors, and a subscore ≥ 8 indites of anxious or depressive state. Participants were divided into two groups, IBD patients with symptoms of anxiety/depression and without. Other questionnaires, including the Inflammatory Bowel Disease Questionnaire (IBDQ), the Composite Autonomic Symptom Score (COMPASS)-31, the Fatigue Severity Scale (FSS) and the Pittsburgh Sleep Quality Index (PSQI) were also asked to be completed at the same time. IBDQ is a disease-specific tool to assess the disease consequences on patient’s quality of life^13^. The COMPASS-31 is a concise and statistically robust instrument to assess autonomic symptoms that provides clinically relevant scores of autonomic symptom severity^14^. FSS and PSQI were used to evaluate the participants’ fatigue and quality of sleep^15,16^.

Patient demographics, disease duration, IBD-related surgery, tobacco use, extra-intestinal manifestations, disease activity scores [Crohn's disease activity index (CDAI) for CD and Mayo score for UC], endoscopic evaluation [Simple Endoscopic Score for Crohn’s disease (SES-CD) and Ulcerative Colitis Endoscopic Index of Severity (UCEIS)], laboratory data [white blood count (WBC), albumin (ALB), erythrocyte sedimentation rate (ESR), C-reactive protein (CRP)] and current medication use were recorded and compared between the IBD patients with symptoms of anxiety/depression and without. IBD-related surgery was defined as patients underwent surgery because of IBD associated stricture, fistula, perforation or cancer at this or previous admissions.

### Statistical analysis

Data was extracted and analyzed with SPSS 22.0 and GraphPad Prism 7.0 software. Continuous variables were presented as median values [interquartile range (IQR)], while categorical variables were presented as percentages. Mann–Whitney U test was used to compare two groups which were continuous variables, and the Pearson chi-square test for categorical variables. Multivariable logistic regression with predictors of symptoms of anxiety and depression in IBD patients were analyzed. Covariates were determined if they reached a threshold level of significance in univariate analyses. Statistically significance was considered achieved for p < 0.05. Of note, we also performed analysis of IBD subtypes.

### Statement of ethics

This study was approved by the Ethics Committee of West China Hospital of Sichuan University and carried out in accordance with the Helsinki Declaration. The purpose and methods of the study were explained to all participants. Written informed consent was obtained from each participant prior to enrollment.

## Results

### Clinical features of IBD patients with symptoms of anxiety/depression

One hundred and thirteen of 341 IBD patients (33.1%) including 120 UC and 221 CD were found to have symptoms of anxiety/depression (Table 1). Prevalence of anxiety/depression was similar between UC and CD (36.7% vs 31.2% respectively; p = 0.336). IBD patients with symptoms of anxiety/depression were older (p = 0.0003), and had lower levels of ALB [35.3 (29.6, 39.7) vs 37.5 (33.1, 42.2), p = 0.0017] compared to those without. Higher level of ESR [49 (31, 71) vs 38 (22, 66), p = 0.0162] and higher frequency of corticosteroid use (44.2% vs 27.6%, p = 0.0032) were observed in patients with symptoms of anxiety/depression. Patients with symptoms of anxiety/depression were more likely to undergo IBD-related surgery (43.4% vs 29.4%, p = 0.0149). Besides, patients with symptoms of anxiety/depression had significantly lower quality of life [125 (97, 140) vs 180 (154, 199), p < 0.0001], which is measured by IBDQ. Increased severity of autonomic symptoms were observed in IBD patients with symptoms of anxiety/depression [26 (15, 39) vs 13 (8.26), p < 0.0001]. Moreover, those patients tended to have higher scores of FSS and PSQI [48 (38, 57) vs 37 (24, 47), 12 (8, 15) vs 6 (4, 9), p < 0.0001, respectively], which meant they were more likely to suffer from poor sleep and more prone to fatigue. (Table 1).

**Table 1:** Characteristics of the entire cohort.

Variable	Cohort (n = 341)	Without symptoms of anxiety/depression (n = 228)	With symptoms of anxiety/depression (n = 113)	*p* value
Disease Type (n), CD/UC	221/120	152/76	69/44	0.336
Women (n, %)	129 (37.8)	85 (37.3)	44 (38.9)	0.8128
Age (years), median (IQR)	33 (14, 77)	31 (24, 40)	37 (27, 51)	0.0003
Disease duration (month)	36 (12, 72)	36 (12, 72)	36 (12, 84)	0.8968
**Tobacoo use, n (%)**				
Never	254 (74.5)	171 (75.0)	83 (73.5)	0.9336
Quitted	48 (14.1)	31 (13.6)	17 (15.0)	
Current	39 (11.4)	26 (11.4)	13 (11.5)	
Extra-intestinal manifestations, n (%)	42 (12.3)	26 (11.4)	16 (14.2)	0.4865
**Laboratory studies**				
WBC (× 10^9^/L), median (IQR)	6.56 (4.82, 8.79)	6.74 (4.78, 8.88)	6.26 (4.87, 8.54)	0.2067
ALB (g/L), median (IQR)	36.6 (32.2, 41.6)	37.5 (33.1, 42.2)	35.3 (29.6, 39.7)	0.0017
CRP (mg/L), median (IQR)	20.85 (6.66, 59.65)	18.05 (4.70, 49.18)	31.3 (9.215, 73.48)	0.0956
ESR (mm/h), median (IQR)	43 (24, 67)	38 (22, 66)	49 (31, 71)	0.0162
**Current therapy**				
Mesalazine, n (%)	134 (39.3)	93 (40.8)	41 (30.6)	0.4801
Corticosteroid, n (%)	113 (33.1)	63 (27.6)	50 (44.2)	0.0032
Immunomodulator, n (%)	143 (41.9)	93 (40.8)	50 (44.2)	0.5615
Anti-TNF, n (%)	72 (21.1)	49 (21.5)	23 (20.4)	0.8882
IBD-related surgeries, n (%)	117 (34.3)	67 (29.4)	49 (43.4)	0.0149
IBDQ, median (IQR)	155 (127, 184)	180 (154, 199)	125 (97, 140)	< 0.0001
COMPASS-31, median (IQR)	19 (10, 32)	13 (8, 26)	26 (15, 39)	< 0.0001
FSS, median (IQR)	42 (27, 52)	37 (24, 47)	48 (38, 57)	< 0.0001
PSQI, median (IQR)	8 (5, 12)	6 (4, 9)	12 (8, 15)	< 0.0001

*CD* Crohn’s disease, *UC* ulcerative colitis, *IBD* inflammatory bowel disease, *WBC* white blood count, *ALB* albumin, *ESR* erythrocyte sedimentation rate, *CRP* C-reactive protein, *TNF* tumor necrosis factor, *IBDQ* inflammatory bowel disease questionnaire, *COMPASS* composite autonomic symptom score, *FSS* fatigue severity scale, *PSQI* Pittsburgh sleep quality index, *IQR* interquartile range.

### Symptoms of anxiety/depression aggravated the severity of IBD

No significant differences were found in age, gender, disease duration, tobacco use between CD patients with symptoms of anxiety/depression and those without (p = 0.1260, 0.0698, 0.8304, 0.7377, respectively). Based on Montreal classification, the disease age, location and behavior were similar between the two CD groups (Table 2). CD patients with symptoms of anxiety/depression tended to have higher CDAI (p < 0.0001), and higher SES-CD (p = 0.0005). White blood cell count or albumin level were not significantly different between the two groups (p = 0.2346, 0.0662, respectively), while inflammatory markers including CRP (p = 0.0190) and ESR (p = 0.0245) were higher in CD patients with symptoms of anxiety/depression versus those without. Furthermore, CD patients with symptoms of anxiety/depression were also more frequently prescribed corticosteroid (42.0% vs 28.3%, p = 0.0434) and were more likely to undergo CD-related surgery (56.5% vs 41.5%, p = 0.0372) (Table 2).

**Table 2:** Characteristics of the CD cohort.

Variable	Without symptoms of anxiety/depression (n = 152)	With symptoms of anxiety/depression (n = 69)	*p* value
Age (years) median (IQR)	31 (23, 40)	34 (24, 42)	0.1260
Women (%)	45 (29.6)	29 (42.0)	0.0698
Disease duration (months), median (IQR)	36 (12, 72)	36 (12, 78)	0.8304
**Tobacco use, n (%)**			
Never	116 (76.3)	55 (79.7)	0.7377
Quitted	16 (10.5)	5 (7.2)	
Current	20 (13.2)	9 (13.0)	
**Montreal age, n (%)**			
A1 (< 17 years)	9 (5.9)	3 (4.3)	0.1965
A2 (17–40 years)	117 (77.0)	47 (68.1)	
A3 (> 40 years)	26 (17.1)	19 (27.5)	
**Montreal location, n (%)**			
L1	44 (28.9)	14 (20.3)	0.1780
L2	22 (14.5)	16 (23.2)	
L3	86 (56.6)	39 (56.5)	
**Montreal behavior, n (%)**			
B1	75 (49.3)	34 (49.3)	0.6513
B2	51 (33.6)	20 (29.0)	
B3	26 (17.1)	15 (21.7)	
Extra-intestinal manifestations, n (%)	18 (11.8)	12 (17.4)	0.2644
Surgeries, n (%)	63 (41.5)	39 (56.5)	0.0372
CDAI, median (IQR)	189.5 (144.0, 280.0)	356.0 (288.5, 436.0)	< 0.0001
SES-CD, median (IQR)	12 (8, 15)	13 (11, 18)	0.0005
WBC (× 10^9^/L), median (IQR)	6.49 (4.76, 8.56)	6.05 (4.75, 7.79)	0.2346
ALB (g/L), median (IQR)	37.1 (33.2, 41.3)	35.4 (31.2, 39.5)	0.0662
CRP (mg/L), median (IQR)	19.6 (6.9, 54.5)	39.8 (10.7, 76.5)	0.0190
ESR (mm/h), median (IQR)	42.5 (24.0, 69.8)	51.0 (37.5, 73.5)	0.0245
Mesalazine, n (%)	45 (29.6)	20 (29.0)	0.9253
Corticosteroid, n (%)	43 (28.3)	29 (42.0)	0.0434
Immunomodulator, n (%)	83 (54.6)	44 (63.8)	0.2017
Anti-TNF, n (%)	43 (28.3)	19 (27.4)	0.9080

*CD* Crohn’s disease, *CDAI* Crohn's disease activity index, *SES-CD* Simple Endoscopic Score for Crohn’s disease, *WBC* white blood count, *ALB* albumin, *ESR* erythrocyte sedimentation rate, *CRP* C-reactive protein, *TNF* tumor necrosis factor, *IQR* interquartile range.

UC patients with symptoms of anxiety/depression were in older age [50 (33, 55) vs 35 (27, 50), p = 0.0037]. No difference in gender (p = 0.0587), disease duration(p = 0.8354), tobacco use(p = 0.5891), disease activity(p = 0.5095), disease distribution(p = 0.6591) and extra-intestinal manifestations(p > 0.9999) was revealed between the two UC groups. UC with symptoms of anxiety/depression had significantly higher Mayo scores [10 (8.5, 11) vs 8 (7, 9), p = 0.0017], higher UCEIS [6 (4.75, 7) vs 4 (3, 5), p < 0.0001], lower albumin levels [34.0 (28.2, 41.7) vs 40.1 (32.5, 44.0), p = 0.0070] and were more likely to undergo UC-related surgery (22.7% vs 5.3%, p = 0.0067). Besides, corticosteroid was also more frequently prescribed (47.7% vs 26.3%, p = 0.0273) for UC patients with symptoms of anxiety/depression. Inflammatory markers such as WBC (p = 0.9177), CRP (p = 0.7314) and ESR (p = 0.1377) were comparable between two groups (Table 3).

**Table 3:** Characteristics of the UC cohort.

Variable	Without symptoms of anxiety/depression (n = 69)	With symptoms of anxiety/depression (n = 44)	*p* value
Age (years) median (IQR)	35 (27, 50)	50 (33, 55)	0.0037
Women (%)	40 (52.6)	15 (34.1)	0.0587
Disease duration (months) median (IQR)	29 (20, 90)	36 (7.5, 84)	0.8354
**Tobacco use, n**			
Never	55 (72.4)	28 (63.6)	0.5891
Quitted	15 (19.7)	12 (27.3)	
Current	6 (7.9)	4 (9.1)	
**Disease activity, n (%)**			
Remission	8 (10.5)	2 (4.5)	0.5095
Mild	10 (13.2)	4 (9.1)	
Moderate	12 (15.8)	6 (13.6)	
Severe	46 (60.5)	32 (72.7)	
**Disease distribution, n (%)**			
E1	8 (10.5)	3 (6.8)	0.6591
E2	22 (29.0)	11 (25.0)	
E3	46 (60.5)	30 (68.2)	
Extra-intestinal manifestations, n (%)	8 (10.5)	4 (9.1)	> 0.9999
Surgeries, n (%)	4 (5.3)	10 (22.7)	0.0067
Mayo score, median (IQR)	8 (7, 9)	10 (8.5, 11)	0.0017
UCEIS, median (IQR)	4 (3, 5)	6 (4.75, 7)	< 0.0001
WBC (× 10^9^/L), median (IQR)	7.21 (5.03, 8.90)	6.90 (5.78, 9.16)	0.9177
ALB(g/L), median (IQR)	40.1 (32.5, 44.0)	34.0 (28.2, 41.7)	0.0070
CRP (mg/L), median (IQR)	11.4 (3.1, 38.2)	16.7 (7.2, 43.0)	0.7314
ESR (mm/h), median (IQR)	27 (16, 47)	41 (24, 66)	0.1377
Mesalazine, n (%)	48 (63.2)	21 (47.7)	0.1259
Corticosteroid, n (%)	20 (26.3)	21 (47.7)	0.0273
Immunomodulator, n (%)	10 (13.2)	6 (13.6)	> 0.9999
Anti-TNF, n (%)	6 (7.9)	4 (9.1)	> 0.9999

*UC* ulcerative colitis, *UCEIS* ulcerative colitis endoscopic index of severity, *WBC* white blood count, *ALB* albumin, *ESR* erythrocyte sedimentation rate, *CRP* C-reactive protein, *TNF* tumor necrosis factor, *IQR* interquartile range.

### Different markers predicted symptoms of anxiety/depression in IBD patients

As shown by multivariable logistic regression, CD-related surgery and CDAI were independently associated with symptoms of anxiety/depression in CD patients, while corticosteroid use was a predictor of symptoms of anxiety/depression in UC patients. (Tables 4 and 5).

**Table 4:** Predictors of symptoms of anxiety and depression in CD patients.

Variable	*p* value	OR	95% confidence interval	
No history of surgery	0.012	0.385	0.183	0.810
CDAI	< 0.0001	1.011	1.007	1.014
SESCD	0.078	1.079	0.992	1.173
CRP	0.464	1.003	0.994	1.013
ESR	0.477	1.005	0.991	1.019
Corticosteroid use	0.162	1.730	0.802	3.732

*CD* Crohn’s disease, *CDAI* Crohn's disease activity index, *SES-CD* Simple Endoscopic Score for Crohn’s disease, *ESR* erythrocyte sedimentation rate, *CRP* C-reactive protein, *OR* odds ratio.

**Table 5:** Predictors of symptoms of anxiety and depression in UC patients.

Variable	*p* value	OR	95% confidence interval	
Age	0.187	0.899	0.767	1.053
No history of surgery	0.462	1.765	0.389	8.017
Mayo score	0.706	0.851	0.369	1.966
Albumin level	0.878	0.950	0.494	1.828
UCEIS	0.421	2.253	0.312	16.292
Corticosteroid use	0.036	3.041	1.076	8.593

*UC* ulcerative colitis, *UCEIS* ulcerative colitis endoscopic index of severity, *OR* odds ratio.

## Discussion

IBD is a relapsing and disabling disease, and its prevalence is increasing worldwide^17–20^. Disease-related stress, financial burden, and some other unrecognized factors may lead to the significantly higher prevalence of symptoms of anxiety/depression in IBD patients compared with that of the general population^9,21–25^. A cohort study found that 40.1% IBD participants met the criteria for diagnosis of depression and 30.6% met criteria for anxiety. However, one-third of participants with depression and two-thirds with anxiety were undiagnosed, especially males^26^. The effects of an untreated mental illness can be devastating and can worsen the disease course of IBD^27^. So, it’s important to timely recognize the mental disorders in the IBD population. To the best of our knowledge, the present study unveiled the state of suffering from symptoms of anxiety/depression in the Chinese IBD population with the largest sample size so far.

Some studies have reported discrepancies as to the prevalence of anxiety and depression and their impact upon IBD subtypes. In a prospective cohort study, depression was associated with an increased risk of CD, but not UC^19^. Other studies showed that psychosocial stress had been purported to play a role in the pathogenesis of both CD and UC^23,24^. Our study demonstrated no significant difference of prevalence of symptoms of A&D between UC and CD. However, it is revealed that the age-adjusted hospitalization rate for CD was 26.9/100,000 and 13.3 per 100,000 for UC^25^, which may explain that CD patients required hospitalization far outnumbered UC patients in our study.

With deep cognition about IBD, quality of life and their mental status are also receiving wide attention^2,10,17,19^. A French cohort showed that a half of IBD patients have reported poor quality of life, severe fatigue^20^. Another review showed that fatigue was reportedly prevalent in 41–48% of patients with IBD in remission and 86% of those in active stage^21^. CD patients with impaired sleep had a twofold increase in risk of active disease at 6 months^22^. However, most of these studies were based on populations from western countries, and the relationship between the above factors were also obscure. The present study was representative of the Chinese IBD population since it was conducted in the largest IBD center of southwest China. In our study, IBD patients with symptoms of anxiety/depression were predisposed to have decreased quality of life and sleep compared with their non-anxiety/depression counterparts. Dysfunction of autonomic nerve and fatigue were more common in participants with symptoms of anxiety/depression. Moreover, we also found that patients with symptoms of anxiety/depression were older, and had lower levels of ALB, which disaccorded to *Navabi*’s study on USA IBD patients^4^.

We identified history of surgery and CDAI as independent predictors of symptoms of anxiety/depression in Chinese CD patients. In contrast, Navabi et al. demonstrated immunomodulator use, history of extra-intestinal manifestations and history of tobacco use as predictors of anxiety/depression in CD, which was inconsistent with our findings^4^. Another study found that anxiety was significantly correlated with female sex, history of perianal disease and perianal surgery in CD patients^28^, which was partly in agreement with our results. These discrepancies may be attributed either to the heterogeneity of participants including races, regions, social cultures, economic conditions, or to the methodology of studies such as standards and protocols. In addition, endoscopic score was found to be the independent risk factors for the symptoms of anxiety/depression. Further studies are needed to define the role of endoscopic score in predicating anxiety/depression for CD.

When it comes to UC patients, corticosteroid use was found to be a predictor of symptoms of anxiety/depression in our study. This is also inconsistent with *Navabi*’s study which revealed that disease duration and immunomodulator use to be predictors of anxiety/depression in UC^4^. Previous research found that initial medical management of IBD was different among countries. For instance, UC patients were more likely to receive immunomodulators in United States^29^. In contrast, moderate-to-severe UC patients in China were often given corticosteroid as the first-line therapy^30^. The fact that most of our participants were moderate-to-severe UC patients may explain that corticosteroid rather than immunomodulator use was an independent predictor. In addition, colectomy is used as the rescue therapy which brings better quality of life for UC^12^, but might be a fuse for severe complications in CD, such as fistula, stricture and short bowel syndrome^31^. This may also explain that the surgery being an independent predictor for symptoms of anxiety/depression in CD rather than in UC.

There are limitations in the present study. Firstly, participants were limited to hospitalized patients, who had worsened disease course compared with outpatients. A study encompassing IBD outpatients showed that the prevalence of depression was 25%, which could not be neglected in the management of IBD^32^. Secondly, this is a cross-sectional, single-center study, despite the largest IBD referral center of the southwest China. Therefore, longitudinal multi-center studies are needed in the future to further strengthen our findings.

In conclusion, this study delineated the impact of symptoms of anxiety/depression on Chinese IBD population and necessitated special attention paid on mental disorders in IBD patients by physicians. Moreover, timely recognition and even psychiatric treatment are very important to improve the prognosis of IBD.
